# Extracellular Vesicles: A Novel Diagnostic Tool and Potential Therapeutic Approach for Equine Osteoarthritis

**DOI:** 10.3390/cimb46110780

**Published:** 2024-11-17

**Authors:** Mohamed I. Elashry, Julia Speer, Isabelle De Marco, Michele C. Klymiuk, Sabine Wenisch, Stefan Arnhold

**Affiliations:** 1 Institute of Veterinary Anatomy, Histology and Embryology, Justus-Liebig-University of Giessen, 35392 Giessen, Germany; julia.speer@vetmed.uni-giessen.de (J.S.); michele.klymiuk@vetmed.uni-giessen.de (M.C.K.); stefan.arnhold@vetmed.uni-giessen.de (S.A.); 2 Clinic of Small Animals, c/o Institute of Veterinary Anatomy, Histology and Embryology, Justus-Liebig-University of Giessen, 35392 Giessen, Germany; isabelle.demarco@vetmed.uni-giessen.de (I.D.M.); sabine.wenisch@vetmed.uni-giessen.de (S.W.)

**Keywords:** mesenchymal stem cells, extracellular vesicles, osteoarthritis, veterinary medicine

## Abstract

Osteoarthritis (OA) is a chronic progressive degenerative joint disease that affects a significant portion of the equine population and humans worldwide. Current treatment options for equine OA are limited and incompletely curative. Horses provide an excellent large-animal model for studying human OA. Recent advances in the field of regenerative medicine have led to the exploration of extracellular vesicles (EVs)—cargoes of microRNA, proteins, lipids, and nucleic acids—to evaluate their diagnostic value in terms of disease progression and severity, as well as a potential cell-free therapeutic approach for equine OA. EVs transmit molecular signals that influence various biological processes, including the inflammatory response, apoptosis, proliferation, and cell communication. In the present review, we summarize recent advances in the isolation and identification of EVs, the use of their biologically active components as biomarkers, and the distribution of the gap junction protein connexin 43. Moreover, we highlight the role of mesenchymal stem cell-derived EVs as a potential therapeutic tool for equine musculoskeletal disorders. This review aims to provide a comprehensive overview of the current understanding of the pathogenesis, diagnosis, and treatment strategies for OA. In particular, the roles of EVs as biomarkers in synovial fluid, chondrocytes, and plasma for the early detection of equine OA are discussed.

## 1. Introduction

Osteoarthritis (OA) is a progressive, degenerative, and multifactorial joint disease that constitutes the most common form of musculoskeletal disorder worldwide [1,2,3]. The disease affects approximately 50 million people in the United States, compared to 100 million reported in Europe, according to [4,5]. As in humans, the disease has a negative impact within veterinary medicine, as reported in horses [6,7], as well as in dogs [8] and cats with hip-joint OA [9]. Achieving satisfactory treatment of damaged articular cartilage has become a major challenge in both human orthopedics and clinical veterinary medicine due to several factors, including the absence of effective pharmacological drugs that provide temporary pain relief and cartilage tissue engineering being insufficient to deliver the optimal therapeutic effect [10]. In this context, the use of mesenchymal stem cells for chondrocyte replenishment in clinical practice has not yet been fully elucidated [11,12]. Moreover, OA is one of the leading causes of lameness in both leisure and sport horses [13,14]. The main symptoms of OA are pain, limited mobility, and stiffness [15]. However, as the disease progresses, the subchondral bone, joint capsule, and adjacent ligaments and muscles may also be damaged [16]. Moreover, in the advanced stage of the disease, OA is accompanied by alteration of the biochemical markers, along with deterioration of the extracellular matrix and the cellular components of the cartilage [17,18]. Reliable treatment options are urgently needed to repair the deterioration of articular cartilage and minimize the subchondral bone damage, which subsequently leads to progressive locomotor dysfunction and joint pain [16]. None of the currently available pharmacological treatments are able to slow the progression of the disease, being limited to symptom management [16]; additionally, some of them cannot reverse the tissue damage, and their application may be accompanied by adverse effects [4]. In addition, the mechanisms of OA’s pathophysiology and development remain poorly understood, involving alterations of several factors and cytokines, such as IL-1β, TNF-α, TGF-β, MMP13, and others [4]. Although the diagnosis is commonly based on clinical examination and radiographic imaging, the insidious nature of the disease causes chronic cartilage damage that becomes less responsive to intrinsic repair due, on the one hand, to the degenerative alterations of the cartilage and loss of type I/II collagen and, on the other hand, to the accumulation of pro-inflammatory cytokines that disrupt the homeostasis of the cartilage matrix [19,20,21]. It has been reported that several factors have been identified as potential causes of OA, including genetic background, aging, and obesity; a clear understanding of OA’s pathogenesis remains to be fully elucidated [4]. A study reported that the progression from joint injury to detectable biological evidence of joint deterioration is difficult to study in humans. The reasons for this include the lack of accurate quantitative assessments of joint injury severity, particularly the biological reaction and tissue alterations, as well as the evaluation of the pathogenesis and the interaction with the other tissues involved in the affected joint. The authors suggested the use of an animal model to detect the biological changes in the course of OA, as well as to evaluate the effects of surgical interference and to optimize the pharmacological treatments [22].

This review provides an overview of the causes, diagnosis, and current therapeutic treatments of OA. We review how to identify and define biomarkers of the disease that can be used to develop rapid and efficient diagnostic tools. We discuss the role of mesenchymal stem cell-derived extracellular vesicles (EVs)/exosomes in modulating the immune response, cellular communication, enhancing autophagy, and inhibiting apoptosis in the joint microenvironment. We also demonstrate the role of MSC secretomes and EVs in OA as potential biomarkers for sensitive and early disease detection, as well as the therapeutic potential of MSC-derived EVs in OA. We selected horses as the large-animal model because the equine stifle joint has a complex anatomical structure similar to that of the human knee, making horses a suitable animal model to evaluate the pathogenesis and clinical findings of OA [23], along with future prospects in equine research regarding the application of EVs for diagnosis and therapy. Thus, EVs offer a novel diagnostic approach, not only because they provide an appropriate biomarker to assess the progression of OA in veterinary medicine through analyzing their contents but because they also aid in understanding the cell-cell and cell-matrix cross-talk to improve OA treatment strategies. 

## 2. Causes and Progression of Equine OA 

In general terms, OA is characterized by multifactorial chronic joint degeneration [24]. Clinical surveys have indicated that around 60% of equine lameness is linked to OA [25,26]. OA results from several overlapping diseases with different causes, but they all lead to similar morphological, biological, and clinical outcomes [27]. The progression of OA depends on both the causative agents and the severity of the condition, starting with articular cartilage and extending to the subchondral bone and surrounding tissues (including ligaments and the synovial membrane) [28]. These changes lead to a series of morphological deteriorations from focal articular degeneration, followed by fibrotic lesions and ulcer formation, to complete loss of the articular surface [29]. In horses, factors such as obesity, aging, genetic predisposition, and trauma contribute to chronic inflammation and long-term joint damage [30]. The metacarpophalangeal and femorotibial joints are common sites of OA in horses [6]. The progression of OA is largely dependent on the inflammatory processes [31,32]. In the course of OA, the inflammatory response due to mechanical stress has been described as altering the balance between anabolic and catabolic processes in the joint [33], thereby causing a predisposition to articular cartilage degeneration, progressive joint damage, and pain [34]. However, an appropriate balance between these processes is essential to maintain cartilage integrity and stop molecular damage during daily activities [33]. Excessive production of pro-inflammatory cytokines such as interleukin 1-β (IL-1β) and tumor necrosis factor-α (TNF-α) drives cartilage matrix degradation, while chemokines (e.g., monocyte chemotactic protein 1, granulocyte-macrophage colony-stimulating factor) and matrix metalloproteinases (MMPs) contribute to the resorption of the subchondral bone [4,35]. It has been reported that a number of inflammatory cytokines play key roles in the development of OA, including IL-1β, TNF-α, IL-6, IL-15, IL-17, and IL-18, as previously reviewed in [36]. One study reported upregulated TNF-α expression in the synovial membrane and the cartilage in equine OA, whereas IL-1β was upregulated only in the cartilage, not in the synovium [37]. Inflammatory cytokines showed different levels of response, which could impact the severity of the disease. While IL-1β plays a key role in both equine and human OA [38], TNF-α is primarily involved in acute OA cases in humans [39]. The inflammatory response in OA disrupts TGF-β’s role in preventing chondrocyte hypertrophy, as IL-1β and other cytokines inhibit TGF-β signaling, leading to cartilage damage [40]. Furthermore, IL-1β has been shown to inhibit the SMAD signaling cascade, which prevents TGF-β receptor synthesis in chondrocytes [41]. In addition, IL-1β stimulates the production of reactive oxygen species (ROS), which not only reduces the expression of oxidative enzymes but also damages articular cartilage [42]. Evidence suggests that pro-inflammatory cytokines such as IL-8, TNF-α, and H_2_O_2_ contribute to chondrocyte hypertrophy, further worsening OA [43,44,45].

## 3. Diagnosis of OA

The basic features of OA can be identified either by the clinical symptom of pain, by physical disability and morphological changes of the joint, or by radiological examination to detect a high bone density, reduced joint space, and the presence of osteophytes. In addition, magnetic resonance imaging (MRI) can be used to detect articular cartilage damage [6]. The process of diagnosing OA has evolved in recent years; characterization of the molecular alterations could provide a diagnostic overview regarding the progression of OA. Based on the detection of biomarkers, one study categorized the sequence of events in the course of knee OA, including pre-, early-, progressive-, and end-stage OA. This facilitated the evaluation of therapeutic applications in a homogeneous group of patients [46]. It has been reported that the measurement of biological markers, including 846 epitopes, chondroitin sulfate, and type II procollagen, can provide an indication of osteochondral fragmentation in synovial fluid and serum in horses [47]. In this regard, the presence of numerous M1 macrophages in the synovium is indicative of pro-inflammatory secretion and high cartilage degradation [48,49]. Furthermore, cytokines and adipokines released from the infrapatellar fat pad indicate local inflammation and are involved in the pathogenesis of OA [50]. Such molecular alterations indicate a developing pathological condition in the joint. Characterization of these biomarkers using a less invasive approach would improve the accuracy of diagnosis. It has been proposed that synovial fluid, blood, and urine are valuable resources for the identification of biomarkers [51]. Although urine and blood samples are easily harvested to detect pathological alterations [52,53], synovial fluid is the optimal resource for identifying molecular biomarkers due to its central role in maintaining the cross-talk between all of the joint compartments [54].

## 4. Current Treatment Strategies for OA

The development of appropriate and effective disease-modifying OA drugs (DMOADs) is currently challenging. The most common treatment options for OA to date are based on non-pharmacological, pharmacological, and surgical approaches to minimize pain and improve joint mobility [55]. However, none of these approaches have shown satisfactory results [56]. Pharmacological therapy is based on the use of nonsteroidal anti-inflammatory dexamethasone (NSAID) to reduce the inflammatory response and temporarily relieve OA-associated pain in horses [57]. Intra-articular steroid administration has been reported in sport horses, including betamethasone, methylprednisolone acetate, and triamcinolone administration, as reviewed in [6]. Intra-articular injection of sodium hyaluronan or polysulfated glycosaminoglycan has been shown to improve the pathology of OA by reducing cartilage fibrillation, which may be a therapeutic choice for OA in horses [58]. Non-pharmacological treatment strategies include physiotherapy to maintain joint function through physical activity [59]. Moreover, electrophysical therapy approaches, including transcutaneous electrical nerve induction, neuromuscular electrical stimulation, pulsed electromagnetic fields, ultrasound, laser therapy, vibration therapy, and shockwave therapy, have previously been described for horses, as reviewed in [60]. Manual therapy, with a focus on the mobilization of specific target joints, can be used to reduce pain and joint stiffness and increase the range of motion in early OA [61,62]. Physiotherapy can improve locomotor and joint performance, as well reducing the accompanying pain and the inflammatory condition. Several therapeutic options can also be used to treat the affected joints, such as ice, heat, electricity, magnetic fields, compression, and movement, as reviewed in [63]. As in humans, exercise plays an essential role in the physiological performance of the joints in young and old horses. It has been reported that mechanical load has a particular impact on cartilage integrity, joint homeostasis, and vascular flow [64]. Exercise has been shown to improve cartilage regeneration and reduce bone remodeling in experimental models of cartilage lesions in horses, as reviewed in [65]. Moreover, laser therapy has been reported as a rehabilitation therapy in which particular care should be taken regarding the application intensity, frequencies, and target tissue depth. The anti-inflammatory effect of laser therapy arises from the stimulation of prostaglandin, which results in vasodilatation; this approach also reduces pain and joint swelling [66]. Therapeutic ultrasound can sufficiently penetrate horse joints, resulting in improvements in tissue metabolism and oxygenation, as well as enhancements in the infiltration of inflammatory cells to the target lesion [60]. Physical methods using focused extracorporeal shockwave therapy (ESWT) have been reported in equine medicine [67]. The use of ESWT has been shown to activate biomarkers related to bone remodeling [68]. Cryotherapy has previously been recommended to reduce the inflammatory response and improve tissue function. A study investigated the effects of induced cryotherapy on the footprint and biochemical components of rat joints. The authors demonstrated that cryotherapy treatment increased the footprint area while reducing the leukocyte count and inflammatory cytokine concentrations in joints in OA patients compared to controls [69]. Similarly, it was determined that short-term cryotherapy could be used as a conservative treatment to relieve pain and improve functional performance in humans with knee OA [70]. The use of magnetic fields in equine physiotherapy has been proposed, with one study showing that applying a magnetic field for one hour every second day may improve cell regeneration and enhance bone symphysis [71]. Along the same lines, an in vitro study investigated the ability of electromagnetic fields to reverse experimentally induced cartilage degeneration in bovines. The authors revealed that a pulsed electromagnetic field could be applied as an adjuvant treatment to protect cartilage from the negative effects of increased inflammatory cytokine concentrations in OA [72]. However, to the best of our knowledge, there are no studies demonstrating the effectiveness of cryotherapy in horses, especially those with advanced OA, and further studies should be conducted to prove the suitability of this method for clinical application. Gene therapy has been proposed as a treatment option in the same context, with one study demonstrating that administration of the interleukin-1 receptor antagonist was able to eliminate the catabolic effect and reduce the progression of OA in horses [73,74]. The use of the aforementioned conventional therapy options is either inadequate or restricted to severe OA due to severe degradation of the articular cartilage, involvement of the subchondral bone, and a reduction in the joint space. Surgical interventions represent the last choice and have been used in human medicine [75,76], involving the repair of the cartilage surface, arthroplasty, or complete joint replacement [77]. Recently, the use of bone marrow- or adipose tissue-derived mesenchymal stem cells (ASCs, BMSCs) has been proposed as a novel therapeutic intervention. It has been reported that intra-articular administration of BMSCs improves prostaglandin E2 levels in synovial fluid, but further studies are recommended to confirm the utility of BMSVs in therapeutic applications [78].

## 5. Inflammatory Responses Trigger Stem Cell Activation During OA

Cell-based therapy has become a promising strategy for tissue regeneration. The isolation and characterization of stem cells provide an alternative tool for the treatment of chronic incurable diseases, including OA and tendinopathy. MSCs have become increasingly promising for addressing the requirements for treating degenerative diseases that are unmet through conventional medicine. Self-renewal and multipotency are the key hallmarks of stem cells, allowing them to act as fundamental units to promote growth, homeostasis, and tissue injury repair. These two important characteristics position stem cells as a reasonable tool for regenerative applications [79,80]. Although MSCs have the ability to differentiate into almost all musculoskeletal cell types [81,82], they also have potent immunosuppressive activities [83,84,85] and the ability to induce vascularization [86]. Therefore, these cells have become an interesting potential choice for tissue engineering applications, especially in veterinary medicine. To date, no conservative therapies have been able to achieve complete tissue repair. Therefore, the application of MSCs in tissue engineering has understandably raised hopes for faster healing and complete repair after musculoskeletal injuries [87]. In addition to their potential differentiation into osteogenic and chondrogenic lineages [88], the use of MSCs has been reported as a potential approach for tendon repair [89]. Despite the use of stem cells as an appropriate resource for cell-based therapeutic applications, several problems have been reported, including loss of guidance cytokines, cellular host rejection, and off-target migration, which collectively could compromise tissue regeneration.

It is well established that the inflammatory response during the development of OA causes chondrocyte and matrix degradation due to the increased release of metalloproteinases, collagenases, PGE2, aggrecans, and free radicals in the joint microenvironment, as reviewed in [6]. A body of evidence has demonstrated the immunomodulatory activity of mesenchymal stem cells [90,91,92]. The key mechanism behind this activity relies on stem cells’ ability to inhibit the proliferation of B and T cells, as well as to decrease the production of pro-inflammatory cytokines and reduce cytotoxicity [93]. Although the ability of MSCs to undergo chondrogenic differentiation has been documented, optimal cartilage repair in OA requires the suppression of cytokines such as IL-1α and TNF-α [94]. It has been observed that MSCs isolated from the synovial membrane have a greater capacity for chondrogenic differentiation compared to BMSCs [95], and these cells were able to improve meniscal cartilage healing in a rabbit model of OA [96]. Similarly, MSCs isolated from the infrapatellar fat pad have proven to be an interesting, easily accessible source for cartilage treatment, as reviewed in [97]. One of the important features of MSCs is their immunomodulatory activity, which helps overcome the negative effects of inflammatory cytokines in the joint microenvironment, thereby facilitating enhanced tissue repair [98]. It has been reported that the action of MSCs is based on the delivery of chemokines and cytokines that not only enable the repair of tissue damage and restoration of normal metabolism but also modulate the inflammatory response. The production of these molecules is relatively dependent on the level of tissue damage and the activity of the host immune system [99]. One study showed that, after MSC treatment, although the cells migrate away from the target site, their chondroprotective and immunomodulatory properties remain active [100]. This effect is probably related to the paracrine effect of MSCs, through which another cell type, such as pericytes, is recruited to enhance tissue repair [101]. Meanwhile, MSCs suppress the proliferation of immune cells at the target site [83]. The chemoattractant also enables the proliferation and differentiation of endogenous progenitors at the site of injury [99]. Identifying the nature of the biological components that regulate networking between the site of injury, immune cells, and MSCs, as well as determining whether these compounds are soluble molecules or transported in small EVs, remains a challenge. Ideally, OA therapy should result in improved joint mobility, no mechanical restriction, and proper integration with healthy adjacent cartilage [102]. In recent years, the use of MSCs from bone marrow or adipose tissue has become the focus of clinical interest in the treatment of OA. Despite the incomplete integration of the cells into the defects, the in situ differentiation of MSCs [103] has chondroprotective effects that inhibit progressive cartilage degeneration by suppressing inflammatory processes [67,103]. A clinical study concluded that the injection of MSCs isolated from adipose tissue into osteoarthritic joints resulted in significant alleviation of lameness symptoms and improvements in movement [104,105].

## 6. Extracellular Vesicles (EVs) Are Potential Biomarkers for the Detection and Diagnosis of OA

### 6.1. Overview of EVs

**Figure 1:**
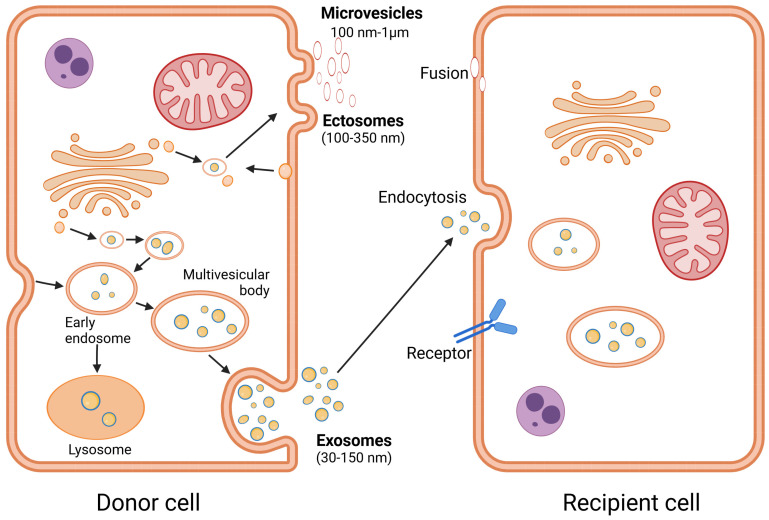
Graphical illustration demonstrating extracellular vesicle biogenesis and secretion and the method by which they target other cells. Exosomes are released from multivesicular bodies (MVBs) via an endosomal process. MVBs bind to the cell membrane to release the exosomes outside the donor cell. Microvesicles are released by budding and detachment from the plasma membrane. The exosomes either internalize the recipient cell via endocytosis, establish ligand-receptor interactions, or fuse into the cell membrane of the recipient cell [119,120].

MSCs are known to play a key role in tissue regeneration. One of the major advantages of stem cells as therapeutic agents is that they can produce biologically active EVs, as EVs can mediate paracrine activity in joint repair in OA [106]. Following the characterization of MSCs as medicinal signaling cells [107], it can be assumed that the therapeutic effects after implantation of MSCs are not based on chondrogenic differentiation in situ but rather on the efficacy of MSC-derived trophic factors released as paracrine-active factors that stimulate regeneration [108]. Although cytokines, growth factors, chemokines, immunomodulatory factors, and signaling lipid mRNAs are involved in this process, regulatory miRNAs are also included within the EVs. EVs play an increasingly important therapeutic role [109]. These vesicles mediate intercellular communication by delivering essential elements such as proteins, lipids, miRNAs, and cytokines to target cells [110,111,112]. Exosomes are vesicles with a diameter of 50–150 nm that are formed intracellularly in microvesicular bodies. They are surrounded by a lipid double membrane and are constricted by the cells. Microvesicles are thus part of the secretome of the cell [113]. It has been reported that EVs/exosomes deliver bioactive products via three different mechanisms (Figure 1). Briefly, (1) surface proteins of exosomes can attach to the membranes of other cells and modulate their activity, (2) the interaction between exosomes and extracellular matrix proteases can release cell receptor activation ligands, and (3) exosomes can integrate into the cell membrane and release their components into the target cell, as reviewed in [114,115]. EVs play a pivotal role in modulating various biological activities and cellular performance. It has been observed that exosomes contribute to the immune response, antigen detection, cell migration and differentiation, and tumor progression [106]. Assessments of the quality and quantity of EVs could facilitate the early detection of pathological alterations in OA, as well as provide an alarm signal to evaluate the severity of the condition and disease progression. These criteria are useful for selecting the optimal therapeutic approach. It was found that EVs play an important role in the pathogenesis of OA; a study revealed that the exosomes isolated from fibroblast-like synoviocytes (FLSs) and chondrocytes play an essential role in OA development, including in inflammatory response and cartilage degeneration, and the impact of these vesicles’ components on the different stages of OA was studied [116]. As part of their function as cargoes of molecular elements in the joint microenvironment, EVs play a pivotal role in mediating intercellular communication, and it has been reported that the assembly of functional Cx43-based channels along the surface of EVs enables the extracellular release of intraluminal contents from these vesicles. This alternative connexin-based mechanism of information transfer to target cells has only been partially elucidated, but according to current knowledge, it has great therapeutic benefits [117]. Exosomal Cx43 has been shown to mediate exosome-based interactions and information transfer between source and target cells [118].

### 6.2. The Biological Characteristics of EVs/Exosomes

Within the group of EVs, exosomes are thought to play a special role in intercellular communication. In addition, exosomes contain small, non-coding RNAs and miRNAs, which mediate their activity and maintain cell-cell communication [121]. MSC-derived exosomes regulate the microenvironment and biological activity of the stem cell with their miRNAs by controlling the balance between proliferation and differentiation [122]. The therapeutic efficacy of EVs has been demonstrated in numerous studies and different cell types. A body of evidence has shown improved tissue regeneration after injury in cardiomyocytes, hepatocytes, and keratinocytes after treatment with exosomes. Furthermore, exosomes have been considered nano-communication vehicles for therapeutic signals due to their low immunogenicity, long half-life, and ability to cross biomembranes [123,124]. Exosomes have been isolated from bodily fluids such as urine [125], milk and blood [126], synovial fluid [127], and platelet-rich plasma (PRP) [128]. The components of the latter source of exosomes, such as growth factors, have shown therapeutic efficacy; one study reported that PRP-derived exosomes improved muscle regeneration after injury in a rat model [129]. 

**Figure 2:**
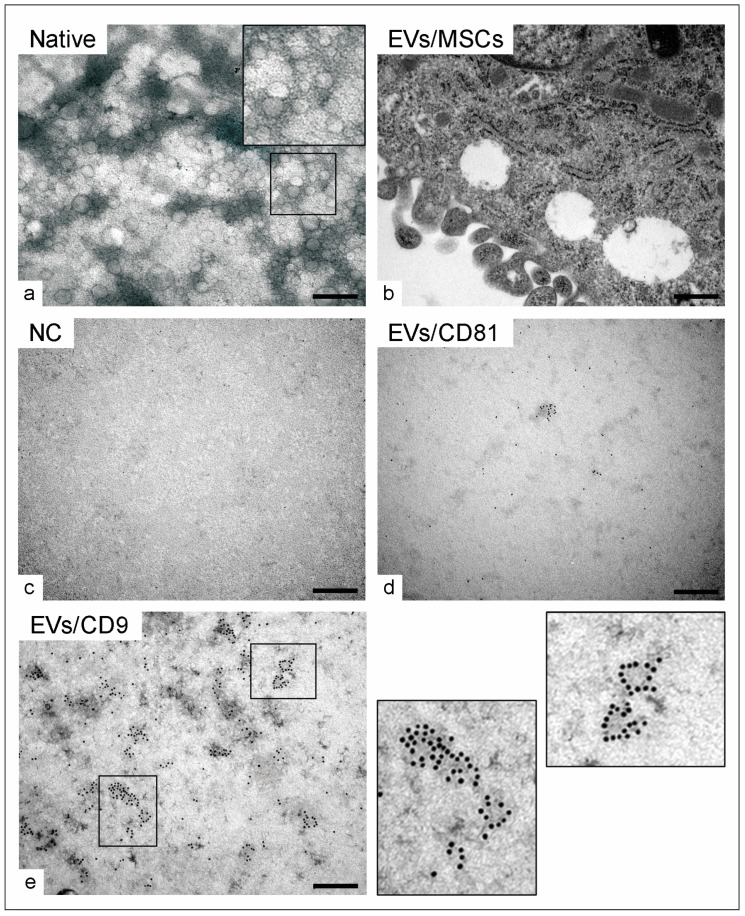
Ultrastructural detection of equine mesenchymal stem cell-derived extracellular vesicles (EVs) using transmission electron microscopy: (**a**) Native EVs/exosomes without immune labeling. (**b**) Mesenchymal stem cell (MSC)-enclosed EVs (white spherical vesicles) in the cytoplasm. EVs were isolated from cell culture supernatant and coupled with 6 nm gold particle antibodies. (**c**–**e**) Immunogold labeling of the specific EV markers, showing (**c**) no primary antibody negative control (NC), (**d**) CD81-positive EVs, and (**e**) CD9-positive EVs. The black squares in (**a**,**e**) indicate a higher magnification of the corresponding area. Scale bars: (**a**,**c**,**d**) 250 nm; (**b**) 500 nm.

Once released into the extracellular milieu, exosomes can be internalized by target cells in both micro- and macroenvironments, thereby contributing to cell-cell communication [130]. EVs/exosomes were isolated via ultracentrifugation at 100,000 rpm for two hours and then processed for transmission electron microscopy analysis, as previously reported by our group [131] (Figure 2a,b). Exosomes can be characterized by the expression of surface marker proteins belonging to the tetraspanin family; these include the surface markers CD9, CD63, and CD81 [132]. Using immunogold labeling, EVs positive for the specific markers CD81 and CD9 could be identified compared to a negative control where primary antibodies were omitted (Figure 2b–d). The presence of exosomes in the cell culture supernatant of MSCs was first described in [133]. In addition, there is evidence for tissue-specific effects of exosomes from different cellular sources [134]. Our recent study showed successful isolation of EVs based on immunogold surface marker detection from equine adipose tissue- or bone marrow-derived stem cells [131,135].

### 6.3. EVs as Potential Biomarkers for OA

Most of the current literature dealing with EVs in the context of OA can be divided into two categories: the first group investigates the potential of EVs isolated from plasma and synovial fluid as biomarkers for the early detection of OA (Table 1), and the second group investigates the potential of EVs as bioactive agents for the treatment of equine OA (Table 2). A biomarker is defined as a characteristic indicator of normal biological or pathological conditions that can be measured using molecular, morphological, or physiological assays. These indicators can be present in either bodily fluids or blood and include any of the following: molecular indicators such as proteins, lipids, and nucleic acids; changes in gene expression; abnormal metabolic byproducts; and changes in tissue architecture, as reviewed in [136]. In a study investigating the response of MSCs to different apoptotic cell lines, the data revealed an upregulated expression and production of stanniocalcin1, indicating the anti-apoptotic potential of MSCs [137]. In the same context, it has been described that co-culture of autologous ASCs with OA-derived chondrocytes counteracts fibrotic activity, including downregulation of MMP13, alkaline phosphatase, Runx2, collagen I/II/VI, and vimentin, along with increased expression of TGF-β [138]. However, these factors are either soluble in the microenvironment or accumulate in EVs, and they have not been fully described. Therefore, tracking these molecular changes by isolating EVs and identifying their components facilitates the establishment of a biomarker for the detection and diagnosis of OA. Similarly, it was found that the quantification of hepatocyte growth factor (HGF), a potent antifibrotic factor in synovial fluid, is indicative of negative progression of OA, which can be used as a biomarker for OA [139]. It has been documented that the chondrogenic differentiation requires the expression of mir193-3p, while upregulation of mir193-3p was detected with chondrogenesis, and expression was not detected in the damaged cartilage. The author reported a low plasma level of exosome-derived miR193-3p in OA patients, indicating that it may be a suitable biological marker for the diagnosis of OA [140].

A study to evaluate EVs as potential biomarkers was conducted using experimentally induced OA as a model for post-traumatic osteoarthritis. After the isolation of EVs using standard procedures, the EVs were characterized according to the criteria of the MISEV guidelines [170]. Plasma-derived and synovial fluid-derived EVs were compared for the expression of relevant microRNAs, including eca-miR-451, eca-miR-25, eca-miR-215, eca-miR-92a, eca-miR-let-7c, eca-miR-486-5p, and eca-miR-23a, along with four snoRNAs (U3, snord15, snord46, and snord58). Bioinformatics analysis revealed that these differentially expressed miRNAs were involved in cell-cycle inhibition, cell-cycle progression, cell proliferation, increased cell viability, and stem cell differentiation in a time-dependent manner. The authors suggested that these miRNAs can be considered not only as potential biomarkers for OA but also as potential therapeutic targets for degenerative joint diseases [146]. In the same context, EVs were characterized by analyzing the microRNA composition in the plasma and synovial fluid of horses with naturally occurring post-traumatic OA. Transcriptome analysis revealed an increase in the percentage of exosomes and a decrease in the percentage of microvesicles compared to horses without post-traumatic OA, as determined via nanoparticle tracking analysis (NTA). Postmortem evaluation of OA was performed in the radiocarpal, middle carpal, and metacarpophalangeal joints. The results of sRNA sequencing revealed that, out of 658 DE miRNAs, only 67 were known, and 591 novel proteins were identified in the plasma EVs of control and post-traumatic horses. Analysis of synovial fluid EVs revealed 805 DE miRNAs, with approximately 74 known and 731 unknown miRNAs for both groups. Surprisingly, 20 DE miRNAs, including miR-19a, miR-29c, miR-132, miR-144, miR-183, miR-185, miR-194, miR-195, miR-199a-3p, miR-200a, miR-200b, miR-219-3p, miR-409-3p, miR-411, miR-499-5p, miR-628a, miR-1301, miR-3200, miR-7177b, and miR-9055, were detected in both plasma- and synovial fluid-derived EVs [148]. The current report suggests that specific markers that can be used for the diagnosis of OA are carried and transferred by EVs.

A proteomic evaluation was employed to detect the early phase of OA in a study that compared the protein cargo of EVs collected from plasma and SF in a time-dependent manner. The proteomic data analysis demonstrated that EV proteins are a more effective predictive tool for assessing OA progression than small non-coding RNAs. Principal component cluster analysis revealed significant discrepancies in the protein cargo between the two EV sources. Concurrently, clusters derived from SF-derived EVs were linked to immune and inflammatory pathways. Conversely, factor analysis of plasma-derived EVs highlighted an increase in proteins that regulate lipid metabolism. Furthermore, protein analysis of SF-derived EVs revealed an association between intermediate filaments and the supramolecular complexes involved in tissue repair. The authors proposed that there is an interaction between time and the development of OA with regard to CRE, NFkB, SRE, and SRF levels. Additionally, EVs derived from the SF of OA patients have been observed to impact chondrocyte proliferation [171]. Similarly, the extent of OA can be assessed in plasma-derived EVs through a combination of Raman and photothermal infrared spectroscopy (RPIS). Plasma samples were collected from thoroughbred racehorses. The optical photothermal infrared imaging (PPII) method demonstrated a high classification rate of 93.4% in distinguishing between EVs derived from osteoarthritic and healthy samples. In contrast, Raman spectroscopy exhibited a lower classification rate of only 64.3%. The higher classification rate achieved with RPIS is based on the detection of increased levels of proteins, lipids, and nucleic acids in EVs derived from osteoarthritic samples. The authors suggested that the former method is a more suitable diagnostic approach than the PPII method for detecting the early stage of OA by utilizing EVs isolated from patient plasma [151]. An evaluation of alterations to the structural elements of the joint could facilitate the early detection of OA and its progression. A study employed EVs as biomarkers in OA to analyze their hyaluronic acid (HA) content in the synovial fluid (SF). For this purpose, EVs were isolated from SF obtained from OA-affected horses following euthanasia. EVs from the contralateral metacarpophalangeal joint were examined in comparison to those from unaffected control horses. The authors were able to distinguish between small and large EVs using NTA and laser scanning microscopy (LSM). Small EVs were not affected by OA, whereas the large HA-containing EVs were markedly reduced. The authors proposed that the reduction in the number of large HA-containing EVs could serve as a diagnostic tool for early-stage OA, preceding the emergence of degenerative evidence through either radiological or clinical symptom assessments [172].

The metabolic performance and components of the joint—for example, fatty acids (FA) and their derivatives, as well as pro- and anti-inflammatory properties—have an impact on the condition of joints, especially during the development of OA. In this context, a recent report compared the FA composition in both SF and SF-derived EVs in equine OA metacarpophalangeal joints using gas chromatography. The FA profiles were modified in both SF and EV-enriched pellets as a consequence of spontaneous OA. The presence of linoleic acid, myristic acid, palmitolic acid, and n-3/n-6 polyunsaturated FAs revealed substantial differences in the SF of OA joints compared to the controls. In EV-enriched pellets, the saturated FAs, including palmitic acid, stearic acid, and behenic acid exhibited alterations indicative of the development of OA. The authors suggested that FA alterations in both EVs and SF represent a valuable biomarker that can impact the pathogenesis of OA. Furthermore, the SF may serve as a potential target for the treatment of OA [156]. Similarly, a recent report employed a combined proteomics and phospholipidomics approach to identify potential biomarkers within SF-derived EVs of equine OA with varying severity, utilizing mass spectrometry. While there was no alteration in the number of EVs in the SF of OA joints, approximately 40 differentially expressed proteins were identified, associated with seven essential trend-setting pathways in OA; these included CD163, myosin regulatory light polypeptide 9, caveolin-1, CD109, ezrin, and moesin [157]. The study indicated that despite the absence of any change in the number of these vesicles, mass spectrometry is vital to detect alterations in EV components in OA at the protein level.

Monitoring the expression of biomarkers in bodily fluids, particularly in SF, represents a valuable means of assessing the progression and development of OA in horses. At present, the diagnosis of OA is typically based on a combination of clinical symptoms and radiographic examination tools. A recent report documented that EVs collected from synovial fluid biopsies using a minimally invasive approach, followed by the targeting of common miRNA expression in exosomes, including miR-504, miR-146a, miR-26a, miR-200c, and miR-210, facilitated the diagnosis of OA [149]. A parallel investigation examined the differential expression of microRNA within synovial fluid and plasma-derived EVs. The analysis demonstrated an increase in the exosome concentration and a reduction in the microvesicle concentration in OA horses compared to healthy controls. Additionally, the expression of miR-144, miR-219-3p, and miR-199a-31 was observed in plasma, while the expression of miR-199a, miR-214, and miR-9094 was detected in synovial fluid-derived EVs. The author concluded that these differentially expressed markers provide evidence of fibrosis, chondrogenesis, and an inflammatory response in post-traumatic OA. The authors concluded that EVs offer a valuable diagnostic tool for understanding the pathology and identifying suitable therapeutic applications for OA in horses [148]. The potential of small non-coding RNAs (sncRNAs) in the synovial fluid as diagnostic markers for early OA was subsequently investigated. The authors identified downregulation of miR-223 in early OA, along with upregulation of miR-23b, let-7a-2, snord96A, and snord13, which were associated with various cellular activities, including inflammation, necrosis, apoptosis, and autophagy. Moreover, mRNA transcripts that target biological cell activities such as proliferation and cellular response were observed to be expressed. The authors suggested that sncRNAs may serve not only as biomarkers for the early detection of OA but also for elucidating the underlying molecular mechanisms involved in the progression of OA [19].

The contents and quantity of EVs, their interaction in the joint microenvironment, and the severity of the condition may be critical, not only for understanding the disease progression but also for selecting the most suitable treatment approach for the patient. It has been demonstrated that the synovial fluid of patients with OA contains higher levels of cytokines, indicative of advanced OA. Furthermore, OA-derived exosomes have been demonstrated to facilitate cartilage degeneration, in addition to an intensified inflammatory response and a reduction in the number of chondrocytes. The author recommended further investigation to detect the composition of EVs as a potential novel diagnostic biological marker for OA [173]. Similarly, the detection of specific molecules could be indicative of OA progression. A study reported that the expression of PCGEM1 was elevated in advanced OA patients compared to those in the early stages, and it also exhibited high expression in the early stages of OA compared to the control group. These findings suggest that PCGEM1 may serve as a valuable biomarker for assessing OA progression and severity [154]. The induction of MSCs using IL-1β leads to the secretion of miR-320c-containing exosomes that enhance chondrocyte proliferation and downregulate MMP13 in OA chondrocytes, indicating pathological improvement [150]. The expression of specific markers in EVs has been demonstrated to impact disease susceptibility, gender differences, and inflammatory progression. It has been demonstrated that the miRNA components of OA exosomes in female patients are estrogen-sensitive and can influence Toll-like receptor signaling. Additionally, the synovial fluid-derived EVs of OA patients modulate the equilibrium between anabolic and catabolic activities in chondrocytes, resulting in the upregulation of catabolic and inflammatory gene expression [145]. Moreover, a study reported that the expression level of miR95-5p was downregulated in exosomes derived from OA cartilage compared to those derived from normal cartilage. Conversely, the treatment of MSCs with articular cartilage-derived miR-95-5p exosomes has been demonstrated to enhance chondrogenic differentiation and matrix expression [174]. These studies indicate that EVs may serve as a valuable source of biomarkers for evaluating the state of inflammation and cartilage deterioration. Moreover, a study was conducted to determine whether cartilage oligomeric matrix protein (COMP) fragments are a potential biomarker for cartilage degeneration in the joints of equines. The authors observed evidence of COMP degradation, fragmentation, and synthesis in the synovial fluid using a monoclonal antibody (14 G4), suggesting that COMP could be a promising marker for OA in equines [175].

A proteomic analysis was conducted to identify the main proteins present in the secretome of articular cartilage, with the aim of mimicking the inflammatory condition observed in OA using 10 ng/mL IL-1β. The authors identified tryptic peptides derived from aggrecan cores, COMP, fibronectin, fibromodulin, thrombospondin-1 (TSP-1), clusterin, cartilage intermediate layer protein-1 (CLIP-1), chondroadherin, and MMP1/3. Furthermore, the induction of inflammation using IL-1β was observed to activate MMP1, MMP3, and TSP-1. Conversely, the level of CLU precursor was found to be reduced, while no change was detected in CLIP-1 or CLU [176]. A similar study monitored the presence of inflammatory cytokines following chondrocyte stimulation with IL-1β for up to 25 days in vitro. The authors identified proteins in the medium that were associated with the inflammatory response, matrix metalloproteinases, acute-phase proteins, IL-6, and complemental components. The authors documented that early-stage OA was characterized by aggrecan and cartilage oligomeric matrix fragmentations after Day 3 and Day 6 post-induction, mimicking the in vivo evidence of early OA. In contrast, the late stage was distinguished by the degradation of the collagenous network after 18 and 22 days [177]. While these studies underscore the alterations in the extracellular matrix secretome, the transfer of these molecules by means of EVs necessitates paying particular attention to the early detection and diagnosis of OA in equines.

A recent study in China investigated a common chronic degenerative disease affecting the joints, known as Kashin–Beck disease (KBD). The objective was to isolate serum- and chondrocyte-derived exosomes for the purpose of evaluating alterations in miRNA. The findings revealed the presence of 53 DE miRNAs in the chondrocyte-derived exosomes. The author employed single-cell RNA sequencing (scRNA-Seq) to identify 115 genes that were targeted by the differentially expressed (DE) miRNA contents of serum- and chondrocyte-derived exosomes. The authors concluded that the analysis of differentially expressed miRNAs using single-cell RNA sequencing is a valuable tool, not only for the diagnosis of the disease but also for the development of therapeutic applications for KBD [178].

A study identified a potential biomarker for the diagnosis of OA based on the presence of autophagy. The study assessed the number and contents of articular cartilage vesicles (ACVs) to determine a correlation between autophagy and OA. The authors reported an increase in autophagy in the ACVs of healthy cartilage, which could be positively induced using rapamycin. In contrast, the ACVs of the OA cartilage exhibited reduced autophagy and a diminished number of ACVs following rapamycin induction. The authors emphasized the importance of autophagy in cartilage regeneration [179]. These findings suggest that the absence of autophagy and the response of ACV numbers to rapamycin may serve as a diagnostic biomarker for OA, distinguishing it from healthy cartilage. 

It is now widely accepted that the transmembrane gap junction protein connexin 43 (CX43) plays a pivotal role in mediating intercellular communication in a multitude of cell types within the musculoskeletal system. Our group has demonstrated the indispensable function of Cx43 in bone remodeling [180], myogenic differentiation [181], and osteogenic differentiation of MSCs, as reviewed in [182]. The expression and the pivotal role of Cx43 in EVs, as well as the potential modulation of EVs by Cx43 in the context of OA, remain incompletely characterized. Evidence suggests that Cx43 expression may serve as a biomarker for the detection and diagnosis of OA in equine patients. It has been reported that overexpression of Cx43 was detected in the chondrocytes of OA patients, increasing the exosomal Cx43 level and causing cell senescence of targeted chondrocytes [183]. Moreover, the senescent chondrocytes exhibited augmented degenerative traits, including the formation of a senescence-associated secretory phenotype (SASP), as well as IL-1β, IL-6, and MMPs. The author suggested a double effect of high levels of Cx43 on exosome activity, influencing cell senescence, inflammatory processes, and cellular reprogramming, which collectively facilitate disease progression. Additionally, the potential utility of Cx43 expression as a biomarker in OA has been proposed [184]. Similarly, a recent report demonstrated that exosomes derived from experimentally induced senescence in ASCs could not protect against cartilage damage or subchondral bone degradation, and they exhibited a lower capacity to polarize macrophages towards an anti-inflammatory phenotype [185]. The existing literature on the role of Cx43 in EV production, the transfer of their components into target cells, and their interaction with cells in the microenvironment of the OA joint is limited. Further investigations into this topic are needed, especially in equine OA.

### 6.4. Immunomodulatory Effect of EVs as a Biological Marker

It is well established that EVs play a role in the inflammatory response during OA, and this response can be used as a biological marker to determine the progression of OA and to modulate the immune microenvironment in the joint. Indeed, exosomes have been demonstrated to contribute to the progression of the pathological profile of OA [186,187]. In the same context, a study reported that adipose-derived MSCs secreted factors that were capable of inhibiting the apoptotic effect of IL-1β on human chondrocytes in vitro [166]. Similarly, a study demonstrated that IL-1β promotes the secretion of exosomes from synovial fluid, resulting in fibroblast cell alteration and subsequent chondrocyte alteration. These alterations are characterized by the upregulation of OA-relative markers, secretion of inflammatory cytokines, extracellular matrix degradation, and antigen presentation, as well as an angiogenic response [188]. The exosomes derived from OA synovial fluid have been observed to promote the secretion of cytokines and chemokines from M1 macrophages [189]. Additionally, incubation of macrophages with OA synovial fluid-derived exosomes has been observed to result in enhanced proliferation and osteoclast formation, independent of the macrophage colony-stimulating factor (M-CSF) and receptor activator of nuclear factor kappa-B ligand (RANKL) [190]. Similarly, a study examined the response of synovial membrane MSCs to the inflammatory cytokine miRNA-140 cultivated in two- and three-dimensional (3D and 2D) culture conditions. The inflammatory condition was induced through the use of IL-1β and TNF-α. The results demonstrated the presence of EVs containing miR-140 in the cells cultivated in a 3D OA condition at 24 and 72 h, respectively. Moreover, the expression of miR-140 was upregulated in the 3D and 2D OA groups at 72 and 120 h, respectively. The authors recommended the utilization of miR-140 as a biomarker for the diagnosis and detection of inflammation in terms of EV tracking for OA in equines [191]. The cross-talk between macrophages and fibroblast-like synoviocytes (FLSs) was examined using a co-culture model, highlighting that lipopolysaccharide activation resulted in the upregulation of IL-1β, IL-6, and ADAMTS4/5 expression in FLSs. The expression of ADAMTS5 was downregulated by DH82 in canine macrophages after incubation with either cells or medium. Moreover, the expression of IL-1β was found to be upregulated following lipopolysaccharide activation in equine FLSs. The authors proposed that macrophages secrete soluble factors that are capable of modulating the inflammatory progression associated with OA in horses [192]. However, the nature of these factors, whether encapsulated in EVs or released directly into the microenvironment, could offer diagnostic value, particularly for the tracking of EVs.

## 7. Stem Cell-Derived EVs/Exosomes: A Potential Therapeutic Choice for OA

The current standard of care for OA is a highly sophisticated and complex approach that primarily relies on anti-inflammatory medications to manage pain and inflammation. This is because no substantial regeneration of cartilage has been observed. Although numerous studies have investigated the use of MSCs in OA, the success rate remains relatively low due to the observation of inflammatory conditions associated with synovitis as a side effect following stem cell injection [193]. The original concept of local MSC differentiation into recipient tissue cells could not be substantiated by the findings of corresponding investigations. EVs/exosomes represent a promising therapeutic tool for chronic degenerative diseases that are unresponsive to traditional medical treatment. The success of MSC treatment was found to be contingent on the secretome of cells, including growth and anti-inflammatory factors [194]. Furthermore, the secretome of MSCs contains EVs. In recent years, research into the potential diagnostic and therapeutic applications of EVs has received considerable attention. The use of EVs as a treatment option has the advantage of being a cell-free approach that does not entail any adverse side effects [195]. The advantages of using EVs/exosomes can be briefly summarized as follows: firstly, EVs isolated from healthy donors or tissue can be used to deliver regenerative products to target lesions; secondly, EVs offer an alternative cell-free approach that excludes the risk of immunoreactivity; and thirdly, it is possible to load EVs with the required bioactive products by activating MSCs in vitro prior to the isolation of EVs from the medium [196]. The impact of stem cell-derived EVs and exosomes on cellular differentiation has been examined extensively. EVs convey signaling molecules that facilitate intercellular communication between donor and recipient cells. A study demonstrated that exosomes derived from osteogenic differentiated mesenchymal stem cells (MSCs) were capable of stimulating osteogenic differentiation in undifferentiated MSCs [197]. Similarly, the isolation of exosomes from the supernatants of various stem cell sources has been demonstrated to prevent the degradation of cartilage and bone in vitro [162]. The presence of inflammatory mediators has been observed to increase the levels of therapeutic miRNAs in exosomes when compared to similar MSC cultures that have not been induced with an inflammatory condition [164]. In addition to their regenerative properties, exosomes have anti-inflammatory and immunomodulatory properties [198]. There is evidence that MSC-derived exosomes both promote the proliferation of chondrocytes and inhibit their apoptosis, mediated via specific miRNAs such as miR-206/GIT1 [166]. It was observed that EVs pre-labeled with a lipophilic stain such as PKH67 were internalized by treated chondrocytes. In the context of the anti-inflammatory effects, a study examined the impact of MSC-derived EVs from bone marrow on the pro-inflammatory cytokines in chondrocytes. The authors observed a significant downregulation of the inflammatory cytokines in the presence of EVs. A similar effect was observed with respect to ADAMTS4 gene expression, suggesting that EVs exert an anti-inflammatory effect on pre-treated equine chondrocytes [135]. In light of the pivotal role of the MSC secretome, a study demonstrated an improvement in the articular cartilage performance following the administration of an MSC secretome for the treatment of OA. This included improvements in chondrocyte function and migration potential compared to MSC therapy alone. The authors recommended further investigation to identify the components of the secretome, such as soluble factors and microvesicles [158].

As in humans, OA causes major locomotor problems in horses; thus, PRP could represent an alternative approach to modulate the inflammatory reaction and the progression of OA in horses, as reviewed in [199]. The intra-articular injection of platelet-rich plasma (PRP) is emerging as a promising therapeutic avenue for patients with OA, as reviewed in [200,201]. It is well established that platelets secrete factors and cytokines that modulate the inflammatory response as well as the regeneration capacity in OA [202]. Platelets contain numerous bioactive chemoattractants that are involved in new matrix formation, tissue angiogenesis, and cellular proliferation at the target site [203]. Specifically, platelets are rich in α-granules [204], which produce chemoattractant factors to promote cell migration and enhance matrix formation [205]. α-granules are a valuable source of factor V fibrinogen, angiogenin, vascular endothelial growth factor, angiostatin, platelet factor IV, metalloproteinases, TNF-α, and IL-1β [206]. In addition, platelets produce platelet-derived growth factor (PDGF), which promotes cell proliferation and proteoglycan production [207]. PRP has a positive impact on reducing the inflammatory response in musculoskeletal lesions, as well as enhancing chondrogenesis, promoting autophagy, and decreasing apoptosis in human OA [208]. Interestingly, however, PRP is a source of exosomes and their numerous bioactive contents. A study demonstrated that PRP-derived exosomes can induce chondrogenic gene expression in OA chondrocytes; the authors assessed the effect of PRP-derived exosomes on experimentally induced OA in rabbits, and the results revealed a reduction in chondrocyte apoptosis, inhibition of TNF-α, and improvement in the OA condition via a mechanism including Wnt/catenin signaling, which suggests a novel therapeutic approach for OA [168]. In a parallel investigation, the impact of PRP on OA chondrocytes was assessed by analyzing chondrocyte proliferation, autophagy, apoptosis, and inflammatory cytokines. The authors observed an increase in chondrocyte proliferation and autophagy following treatment with PRP from healthy donors. PRP treatment was observed to reduce cell apoptosis and induce a dose-dependent reduction in the expression of MMP3, MMP13, ADAMTS-5, IL-6, and COX-2. In contrast, the expression of TGF-β, aggrecan, collagen II, and TIMPs was upregulated, as were the intracellular levels of IL-4, IL-10, and IL-13 [208]. Nevertheless, the beneficial impact of PRP on OA could be attributed to the release of soluble factors directly into the plasma, or to the delivery of microvesicles. Therefore, elucidating the nature of these secretory products could enhance the therapeutic potential of PRP, particularly in terms of isolating and characterizing EVs from PRP.

In a more sophisticated approach, MSCs were isolated from adipose tissue and selected for high expression of α10β1 integrin using MACS cell sorting. Following the selection procedure, the cells were injected into an experimentally induced osteochondral lesion in the carpal joints on Day 18 after lesioning. EVs derived from SF were isolated using ultracentrifugation. The authors observed the presence of a multitude of proteins, including serine endopeptidase activity, complement activation, and collagen extracellular matrix, following MSC treatment. The authors concluded that EVs may play a role in mediating the effects of cell therapy due to altered joint homeostasis, highlighting the therapeutic mechanisms of MSC-based OA therapy [209]. This approach provides valuable insights into the analysis of the effects of OA treatments, with or without cells, via the protein equipment of EVs in joint fluid.

In an experiment, equine articular chondrocytes were isolated from the carpal or femoral condyles and cultivated in the presence of MSC-conditioned media in vitro. The expression of markers for cartilage and cell proliferation was examined in equine articular cartilage cells, either in monolayer cultures or in three-dimensional organoid cultures. The authors documented that the treatment of articular cartilage cells with the conditional medium resulted in a positive impact on cartilage markers and cell migration. The authors of this study concluded that cell-free therapy (whereby the conditioned medium is administered, including EVs) represents a promising approach for the treatment of OA. Nevertheless, additional analysis is necessary to elucidate the mechanisms of action [158].

The method of isolation and purification may influence the therapeutic potential of EVs. It was previously demonstrated that MSC-derived EVs isolated using membrane affinity capture (MAC) exhibited a more pronounced beneficial impact on equine articular cartilage (eAC) cells cultivated as cartilaginous organoids in comparison to EVs isolated via size-exclusion chromatography (SEC). An improvement of eAC was observed, including the synthesis of a hyaline-like matrix, an increase in the expression of type IIB and type I collagen, a modulation of type X collagen, and an alteration in Htra1 and PCNA levels. Subsequently, there was an increase in the production of MMP1 and MMP3. The authors concluded that the reason why MAC-purified exosomes led to a superior improvement in the eAC phenotype compared to SEC-purified EVs may be attributed to the enhanced sample purity achieved through MAC purification [159], as the low vasculature and oxygen concentration in the cartilage microenvironment impedes the regeneration capacity. A study was conducted to investigate the effects of BM-MSC-derived exosomes overexpressing miR-140-3p on chondrocytes that had been previously induced with the inflammatory cytokine IL-1β and maintained under a low oxygen concentration. The authors reported that exosome treatment improved the proliferative and migratory ability of inflammatory chondrocytes and increased chondrogenesis. Furthermore, the healing activity of inflammatory chondrocytes was mediated via the positive effect of hypoxia on miR-140-3p. The authors suggested that hypoxia improves the regenerative ability of chondrocytes, perhaps by regulating the miR-140-3p in BM-MSC-derived exosomes, which improves the healing ability in OA [161].

## 8. Research and Future Prospects of EVs in Veterinary Medicine

In the field of veterinary medicine, investigations into EVs represent a relatively novel and rapidly expanding area of research. The number of publications on EVs is increasing annually, with studies exploring the diverse roles of EVs in animal physiology and pathology, as well as their potential applications as diagnostic biomarkers and in therapy. The most appealing aspects of EVs are their natural biocompatibility, biodegradability, and low immunogenicity, which make them an attractive option for drug delivery approaches [210]. The tremendous increase in the number of EV studies in veterinary medicine mainly reflects the current global concerns under the concept of “one health”, such as the need for high productivity and better welfare in animal production, control of emerging infectious diseases and zoonoses, and environmental monitoring [211,212,213]. Understanding the performance of EVs in large animals’ physiology and pathology could be of great importance for the overall outcomes of EV research targeting OA. Indeed, animals and humans have similar physiology and share diseases such as cancer and musculoskeletal diseases (e.g., OA), which also makes animals good spontaneous and inducible models for research in human medicine [214,215]. It has been reported that OA is common in both horses and humans and has a similar pathogenesis; thus, horses may serve as a large-animal model to confirm theoretical and clinical findings from human orthopedics [216]. This is made even more important by the fact that most studies on the diagnosis and treatment of OA have so far been carried out in small-animal models with limited transferability to human patients [217]. At present, the available literature regarding the identification, diagnostic value, and therapeutic benefit of EVs or exosomes in equine veterinary medicine is scarce. Thus, it is worthwhile to take a closer look at the diagnostic options and therapeutic opportunities presented by the use of EVs/exosomes in equine medicine for the diagnosis and treatment of OA. In addition, the important role of MSC-derived EVs/exosomes in orchestrating the inflammatory response and cartilage regeneration represents a potential cell-free therapeutic tool for OA. However, the lack of essential information impacts the search for an optimal isolation method, the identification of biomarkers relevant to OA progression, and the suitable conditions for EV storage, transfer, and injection for therapeutic use, which require further investigation in equines. EVs’ viability and biocompatibility, either in hydrogels or loaded in microcarriers, as well as their potency following therapeutic application, remain an interesting hot topic, not only for equine OA but also for human OA.

## 9. Conclusions

The potential of EVs for the diagnosis and treatment of joint diseases in equine orthopedics remains largely unexplored. In veterinary medicine, only a limited number of studies have thus far addressed this significant and promising research area. Initial findings suggest that extracellular vesicles (EVs) obtained from blood plasma and synovial fluid may serve as an early diagnostic tool due to their rich composition of specific microRNAs (miRNAs) and proteins that play a pivotal role in regulating the inflammatory response, cell proliferation, autophagy, and apoptosis associated with OA. The identification of changes in biomarkers within EVs enables the early detection of OA and the selection of the most appropriate treatment strategy. Concerning the therapeutic potential of EVs, the data derived from in vitro studies and preclinical investigations suggest that EVs exert anti-inflammatory and regenerative effects. In light of these findings, EVs represent a promising potential therapeutic agent for the treatment of OA. The efficacy of EVs has been demonstrated in some preliminary, early-stage, and pre-clinical OA studies. In addition, the use of cell-free therapeutic bioproducts avoids the problems and side effects that have been repeatedly reported with the use of MSCs. Therefore, further studies are urgently needed to standardize the isolation methods and to characterize the constituents of EVs more precisely, such that EVs can be offered as a safe therapeutic option as one component of the mesenchymal stem cell secretome.

## Figures and Tables

**Table 1:** Contents of EVs and their functions in the context of OA.

Composition	Response to OA/Function	Reference
**(1) miRNAs** miR-372 miR449a-5p	Increased expression in OA to protect chondrocytes from apoptosis Upregulated in advanced OA patient undergoing total knee replacement	[141,142]
miR-126-3p	Decreases with OA; suppresses apoptosis, inflammation, and osteophyte formation in chondrocytes	[143]
miR-500b, miR-720, miR-4454, miR-199b-5p miR-3154	Upregulated after IL-1β induction	[144]
miR-504-3p	Upregulated with OA	[145]
eca-miR-451, eca-miR-25, eca-miR-215, eca-miR-92a, eca-miR-let-7c, eca-miR-486-5p, and eca-miR-23a; snoRNAs	Involved in cell-cycle inhibition, cell-cycle progression, cell proliferation, and differentiation; potential biomarkers for OA	[146]
miR-372-3p	Upregulated with OA	[141]
miR-449a-5p	Increased expression in chondrocytes following IL-1β treatment	[142]
miR-155-5p	Upregulated with potential induction of an TNF-α-based inflammatory response	[147]
mir193-3p	Upregulated with chondrogenesis; expression was not detected in the damaged cartilage of OA patients	[140]
miR-19a, miR-29c, miR-132, miR-144, miR-183, miR-185, miR-194, miR-195, miR-199a-3p, miR-200a, miR-200b, miR-219-3p, miR-409-3p, miR-411, miR-499-5p, miR-628a, miR-1301, miR-3200, miR-7177b, and miR-9055	Detected in plasma- and synovial fluid-derived EVs of horses with OA	[148]
miR-199a, miR-214, and miR-9094	Differentially expressed, indicating fibrosis, chondrogenesis, and an inflammatory response in post-traumatic OA	[148]
miR-504, miR-146a, miR-26a, miR-200c, and miR-210	Targeting their expression could be diagnostic for OA	[149]
miR-320c	Enhances chondrocyte proliferation, downregulates MMP13 in OA chondrocytes, and indicates pathological improvement	[150]
miR-140	Upregulated in OA; can be used as a biomarker	[151]
miR-146a	Strong expression in early OA cartilage; plays a role in OA cartilage pathogenesis.	[152]
miR-145, miR-221	Inhibits inflammation and improves chondrogenesis of the OA joint	[153]
HULC	The amount decreases with OA progression	[141]
lncRNA	Osteoarthritis marker	[154]
PVT1	Regulates OA progression via modulating HMGB1/T1r4/NF-kB signaling	[155]
PCGEM1	Osteoarthritis marker	[154]
**Fatty acids (FAs)** palmitic acid, stearic acid, and behenic acid	FA alteration level is indicative of OA development, providing a valuable biomarker for OA pathogenesis	[156]
**Proteins** CD163, myosin regulatory light polypeptide 9, caveolin-1, CD109, ezrin, and moesin	Differentially detected in the EVs of horses with OA	[157]

**Table 2:** Using EVs for OA management.

Source of EVs	Species	Conclusion	Reference
BMSCs	Horse	Improves the articular cartilage performance including chondrocyte function and migration potential compared to MSC therapy alone	[158]
BMSCs	Horse	Exosomes primed with equine proinflammatory cytokines promote articular cartilage regeneration	[159]
BMSCs	Rat	Reduces the effect of OA through promoting phenotypic transformation of synovial macrophages from M1 to M2	[160]
BMSCs	Rat	Treatment with miR-140-3p-overexpressing exosomes under hypoxia increases the survival rate, migration of inflammatory chondrocytes, and chondrogenesis	[161]
BMSCs	Horse	Delivers an anti-inflammatory effect to artificially inflamed autologous chondrocytes	[135]
BMSCs	Mouse	Demonstrates chondroprotective and anti-inflammatory properties in a murine model of OA	[162]
ASCs	Human	Exosomes overexpressing miR-145 and miR-221 reduce the inflammation response and improve cartilage regeneration	[153]
ASCs	Mouse	Exosomes of TNF-α preconditioned MSCs ameliorate the pathological changes in the joint of OA mice	[163]
ASCs	Human	Inflammatory priming enhanced the healing capacity of ASC-derived EVs in an animal model of OA	[164]
MSCs	Human	Improves cartilage regeneration, restores tissue injuries, and improves the joint performance of OA patient	[165]
MSCs	Human	Improves chondrogenesis and attenuates the negative effect of proinflammatory cytokines	[166]
SMSCs	Mouse	Exosomes overexpressing miR-155-5p enhance the physiological performance of chondrocytes, attenuate apoptosis, and modulate matrix production in an OA mouse model	[167]
PRP	Rabbit	Promotes OA cartilage repair by increasing proliferation and inhibiting apoptosis of chondrocytes	[168]
UCMSCs	Human	Improves cartilage repair in an OA rat model	[169]

## Data Availability

The data presented in this study are available upon reasonable request from the corresponding author. The data are not publicly available due to privacy concerns.

## Abbreviations

MSCs	Mesenchymal stem cells
ASCs	Adipose tissue-derived mesenchymal stem cells
BMSCs	Bone marrow-derived mesenchymal stem cells
IL-1β	Interleukin 1-β
TNF-α	Tumor necrosis factor-α
MMPs	Matrix metalloproteinases
ROS	Reactive oxygen species
TGF-β	Transforming growth factor-β
NSAID	Nonsteroidal anti-inflammatory dexamethasone
HA	Hyaluronic acid
PGE2	Prostaglandin E2
PRP	Platelet-rich plasma
SncRNA	Small non-coding RNA
COMP	Cartilage oligomeric matrix protein
TSP-1	Thrombospondin-1
CLIP-1	Cartilage intermediate layer protein-1
M-CSF	Macrophage colony-stimulating factor
RANKL	Receptor activator of nuclear factor kappa-B ligand
TIMPs	Tissue inhibitors of matrix metalloproteinase
HGF	Hepatocyte growth factor
HULC	Highly upregulated in liver cancer
SMSCs	Synovial mesenchymal stem cells
UCMSCs	Umbilical cord mesenchymal stem cells
